# Effect of Cementite on the Hydrogen Diffusion/Trap Characteristics of 2.25Cr-1Mo-0.25V Steel with and without Annealing

**DOI:** 10.3390/ma11050788

**Published:** 2018-05-12

**Authors:** Yan Song, Zelin Han, Mengyu Chai, Bin Yang, Yilun Liu, Guangxu Cheng, Yun Li, Song Ai

**Affiliations:** 1 School of Chemical Engineering and Technology, Xi’an Jiaotong University, Xi’an 710049, China; songyan1211@mail.xjtu.edu.cn (Y.S.); hanzelin@stu.xjtu.edu.cn (Z.H.); chaimengyu929@stu.xjtu.edu.cn (M.C.); yangbinxjtu1993@163.com (B.Y.); 2 School of Aerospace, Xi’an Jiaotong University, Xi’an 710049, China; yilunliu@mail.xjtu.edu.cn

**Keywords:** hydrogen embrittlement, 2.25Cr-1Mo-0.25V, hydrogen diffusion, hydrogen trap, electrochemical permeation method

## Abstract

Hydrogen embrittlement (HE) is a critical issue that affects the reliability of hydrogenation reactors. The hydrogen diffusivity/trap characteristics of 2.25Cr-1Mo-0.25V steel are important parameters mainly used to study the HE mechanism of steel alloys. In this work, the hydrogen diffusivity/trap characteristics of heat-treated (annealed) and untreated 2.25Cr-1Mo-0.25V steel were studied using an electrochemical permeation method. The microstructures of both 2.25Cr-1Mo-0.25V steels were investigated by metallurgical microscopy. The effect of cementite on the hydrogen diffusivity/trap mechanisms was studied using thermodynamics-based and Lennard–Jones potential theories. The results revealed that the cementite located at the grain boundaries and at the interfaces of lath ferrite served as a kind of hydrogen trap (i.e., an irreversible hydrogen trap). In addition, hydrogen was transported from ferrite to cementite via up-hill diffusion, thereby supporting the hypothesis of cementite acting as a hydrogen trap.

## 1. Introduction

The increasing demand for higher efficiency refining equipment in the petroleum refining industry has resulted in hydrogenation reactors to cope with harsher operating conditions (e.g., higher temperature and pressure conditions). Owing to its good thermal-mechanical properties, 2.25Cr-1Mo-0.25V steel has been developed and used for manufacturing hydrogenation reactors. However, hydrogen can be inserted into the steel during the manufacturing and operating processes. This may lead to the degradation of the mechanical properties of the 2.25Cr-1Mo-0.25V steel, (i.e., hydrogen embrittlement (HE) [1,2]), and may also lead to hydrogen-induced cracking (HIC) [3,4] during the operating process. These will gravely affect the reliability of the reactors. Much work has been done to study the HE of 2.25Cr-1Mo-0.25V steel. Garcia et al. [1] analyzed the effect of HE on the tensile properties of three different grades of CrMoV steel by the small punch experiment, indicating that CrMoV steel has a high susceptibility to HE, and the tensile properties are relative to the different grades of the CrMoV steel. Song et al. [2] studied the effect of HE on the fracture toughness of 2.25Cr-1Mo-0.25V steel and the welds after annealing by a three-point bending experiment and found that the fracture toughness for the steel and welds significantly decrease under hydrogen-charged conditions, and the steel shows a superior resistance to HE than the welds. Meanwhile, there are two mechanisms for HE in metallic materials: (i) hydrogen-enhanced decohesion model (HEDE) [5,6], (ii) hydrogen-enhanced localized plasticity model (HELP) [7].

The diffusivity of hydrogen and the hydrogen trap (i.e., irreversible or reversible traps) are two important parameters which are mainly used to study the HE mechanism of steel alloys. These two parameters are usually obtained experimentally via an electrochemical permeation method. Numerous studies have been carried out on this topic. S. Frappart et al. [8] studied the diffusion of hydrogen and the segregation into a Fe-C-Mo martensitic high-strength low-alloy (HSLA) steel using an electrochemical permeation test. These authors characterized the microstructure by optical microscopy and transmission electron microscopy and defined various degrees of potential trap sites, namely, grains and lathes boundaries, precipitation states, and dislocations. Q. Cui et al. [9] studied the effect of nanosized NbC precipitates on hydrogen diffusion through X80 pipeline steel. S. Frappart et al. [10] also studied hydrogen solubility, diffusivity, and trapping phenomena of tempered Fe-C-Cr martensitic steel under various mechanical stress states. These authors found that the local elastic distortion associated with hydrogen atoms in lattice sites and residual vacancies seemed to affect the hydrogen lattice concentration, while the elasticity and the micro-plasticity did not affect the apparent diffusion coefficient; Therefore, an investigation on the diffusivity of hydrogen and the hydrogen trap (i.e., irreversible or reversible) characteristics of 2.25Cr-1Mo-0.25V steel can significantly contribute to the optimal design and reliability assessment of hydrogenation reactors. However, to the best of our knowledge, the diffusivity of hydrogen and the hydrogen trap (i.e., irreversible or reversible) characteristics of 2.25Cr-1Mo-0.25V steels have been rarely studied.

In addition, the effect of microstructures of materials on the hydrogen diffusion and trap have been studied. F. Huang et al. [11] studied the effect of microstructure and inclusions on the hydrogen trapping efficiency of X 120 pipeline steel and found that the microstructure, especially that containing granular bainite and M/A microconstituents, and inclusions play crucial roles in hydrogen trapping. G.T. Park et al. [12] studied the effect of the microstructure on the hydrogen trapping efficiency and hydrogen induced cracking of line pipe steel. These authors revealed that the key microstructures (i.e., degenerated pearlite (DP), acicular ferrite (AF), and bainite (B)) affected both hydrogen trapping and hydrogen diffusion. Thus, AF and B acted as reversible trapping sites, with the hydrogen trapping efficiency increasing in the order: DP, B, and AF. These studied shows that the cementite is a crucial phase when it comes to hydrogen diffusion and trapping. However, the mechanism of cementite on the hydrogen trapping has been rarely studied. In this work, we tried to explain this mechanism based on the diffusion thermodynamics theory [13] and Lennard–Jones potential theory [14].

In this work, the hydrogen diffusivity and trap characteristics of 2.25Cr-1Mo-0.25V steels heat-treated (annealed) and untreated were studied using an electrochemical permeation method. The microstructures of these annealed and untreated 2.25Cr-1Mo-0.25V steels were investigated by microstructure characterization with the aid of optical microscopy (OM) and scanning electron microscopy (SEM), and we found differences in the microconstituent and grain size characteristics of both steels. The mechanism of cementite on the hydrogen diffusion and trap was studied using thermodynamics-based and Lennard–Jones potential theories, and it was found that the diffusion of hydrogen from ferrite to cementite is up-hill diffusion, which resulted in cementite trapping hydrogen atoms.

## 2. Experimental Method

### 2.1. Materials

**Figure 1:**
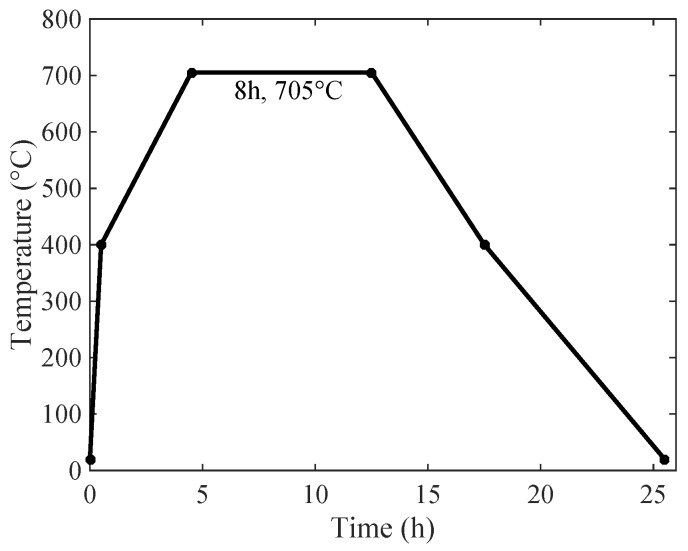
Heat treatment (annealing) procedure [2].

2.25Cr-1Mo-0.25V steel was provided by ArcelorMittal Company. The steel was treated by normalizing (910 °C/Furnace cooling) and tempering (720 °C/Air cooling) heat treatments, then two of the steel plates (i.e., thickness: 98 mm, width: 320 mm) were joined by the narrow-gap welding process. CM-A106HD wire was used for Shielded Metal Arc Welding for root welding. Submerged Automatic Arc Welding was performed for the remaining passes with filler metal (i.e., US-521H) and flux (i.e., PF500). The travel speed, welding current, and arc voltage were maintained at 22 mm/min, 500 A, and 32 V, respectively. Finally, the post-weld heat treatment (PWHT, i.e., annealing) was performed by Lanzhou LS Heavy Equipment Co., Ltd. (Lanzhou, China) and the annealing process is shown in Figure 1. The 2.25Cr-1Mo-0.25V steel used in this work came from the base metal of the welded joint, and its chemical composition is shown in Table 1. The untreated steel (i.e., without PWHT) was denoted as raw steel (RS), while the annealed material (i.e., with PWHT) was denoted as annealed steel (AS).

### 2.2. Hydrogen Electrochemical Permeation Experiments

#### 2.2.1. Preparation

This step included mainly specimen machining and electroplating steps. The specimens were machined to square membranes of 33 mm in size and with an initial thickness of 1.5 mm. The presence of surface oxides can affect surface absorption and desorption processes during the hydrogen electrochemical permeation experiment, leading to significant experimental errors [15]. To avoid this, oxides were removed from both sides of the specimens by grinding them with abrasive papers from 320 to 1000 grit. After grinding, the specimens were rinsed with deionized water, degreased with acetone for 5 min in an ultrasonic cleaner, rinsed with ethanol, and dried with cold air. Finally, the specimens were electroplated with nickel in order to avoid corrosion by the solution and ensure the permeation of hydrogen atoms. Prior to the electroplating process, the specimens were stroked in a strong acid solution to activate the surface and therefore ensure a close adhesion between nickel and the substrate. The electroplating process used the galvanostatic method. The current density and the charging time were 4 A·dm^−2^ and 350 s, respectively.

#### 2.2.2. Hydrogen Electrochemical Permeation Tests

**Figure 2:**
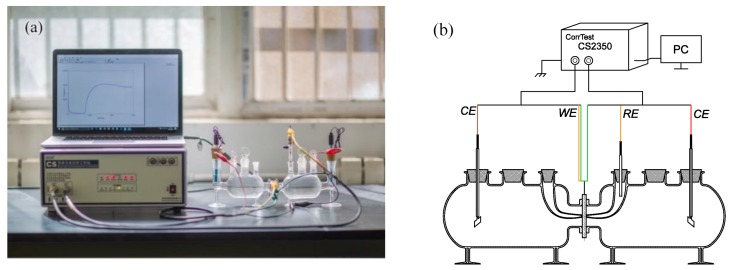
Hydrogen electrochemical permeation device: (**a**) real photo and (**b**) schematic. CE indicates counter electrode, WE indicates working electrode, and RE indicates reference electrode.

Hydrogen electrochemical permeation tests were performed following the method developed by Devanathan and Stachurski [16]. This method used Devanathan–Stachurski cells containing two cells and auxiliary glassware, as shown in Figure 2. The specimen was located and sealed between the two cells, with an exposed area of 2.27 cm^2^ to each cell. The right cell (anodic or hydrogen oxidation cell) was filled with a deaerated 0.5 mol·L^−1^ NaOH solution. The left cell (cathodic or hydrogen charging cell) was filled with 0.5 mol·L^−1^ H_2_SO_4_ and 0.5 g·L^−1^ Na_4_P_2_O_7_ solutions. Na_4_P_2_O_7_ acted as poisoning agent to prevent recombination of hydrogen atoms into hydrogen molecules [9]. Prior to each test, the specimen was hydrogen discharged via anode potentiostatic polarization at a potential of 200 mV vs. a Hg/HgO reference electrode. When the current density decreased below 1 μA, the discharging process was completed and the charging test started. During the test, a constant cathodic hydrogen charging current density of 30 mA·cm^−2^ was applied to the cathode cell for driving hydrogen atoms into the specimen. While the hydrogen atoms diffused and reached the surface of the specimen in the anode cell, the potentiostatic polarization of the specimen (i.e., discharging process) ensured complete oxidation of the hydrogen atoms. Simultaneously, the anodic hydrogen oxidation current was recorded by a CorrTest CS2350 electrochemical workstation (CORRTEST, Wuhan, China). The discharging and charging tests indicated above were repeated on the same specimen to show the effect of different types of hydrogen traps on the specimen. All tests were conducted at ca. 30 °C.

### 2.3. Analysis of the Hydrogen Permeation Tests

The hydrogen flux $J_{\infty }$ (mol·m^−2^·s^−1^) through the specimen was determined by measuring the steady state hydrogen oxidation current density, $i_{\infty }$ with the following expression [12,17]:

(1) $$J_{\infty }=\frac{i_{\infty }}{AF}$$

where A is the exposed area of the specimen (2.27 cm^2^) and F is the Faraday’s constant (96,485.33 C·mol^−1^) The hydrogen diffusivity D (cm^2^·s^−1^) can be calculated by the time lag and breakthrough methods [16,18]:

(2) $$D_{eff}=\frac{L^{2}}{6t_{L}}$$

(3) $$D_{app}=\frac{L^{2}}{15.3t_{b}}$$

where L is the thickness of the specimen (1.5 mm), $D_{eff}$ is the effective hydrogen diffusivity at the time lag $t_{L}$, which corresponded to the time required for the anodic current to reach 63% of its steady state value. $D_{app}$ is the apparent hydrogen diffusivity at the breakthrough time $t_{b}$, which is the intersection of the tangent with the anodic current curve and the time axis when the first diffused hydrogen was oxidized in the anode cell. The subsurface hydrogen concentration was defined according to the Fick’s first law as follows:

(4) $$C_{0}=\frac{J_{\infty }L}{D_{eff}}$$

### 2.4. Microstructure Characterization

All specimens used in this work were ground with abrasive papers up to 1500-grit and polished with 1 μm diamond pastes. Subsequently, the specimens were etched with Nital (a mixture solution of 5% nitric acid and ethanol) and rinsed with ethanol immediately. The microstructure was observed using an optical microscope (ECLIPSE, Melville, LA, USA) and SEM (TESCAN, Brno-Kohoutovice, Czech Republic).

## 3. Results

### 3.1. Microstructure Characterization

**Figure 3:**
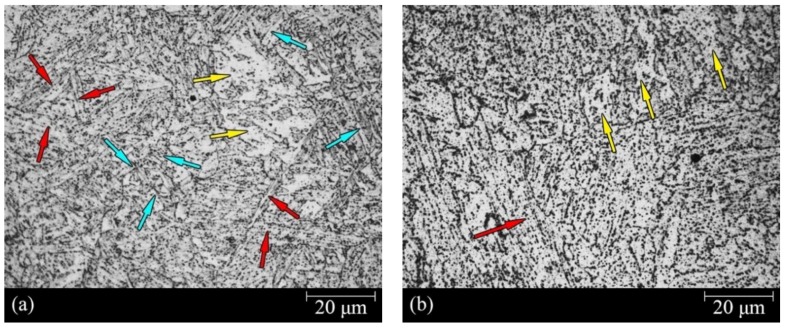
Optical microscopic images of: (**a**) raw steel (RS) and (**b**) annealed steel (AS). Red arrows indicate acicular ferrite, yellow arrows denote quasi-polygonal, and blue arrows indicate lath ferrite.

**Figure 4:**
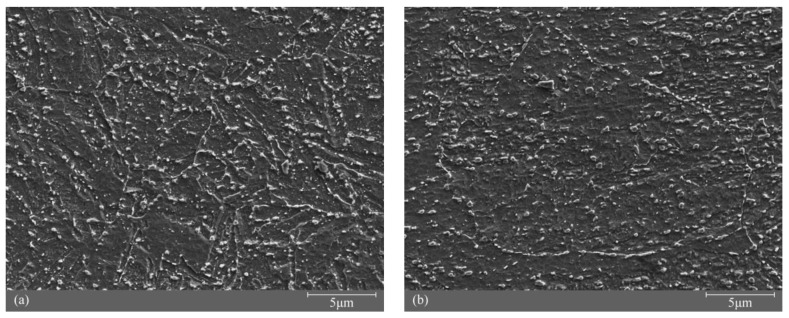
SEM images of: (**a**) RS and (**b**) AS.

Figure 3 shows the typical microstructures of RS and AS. Although both showed a main structure of granular bainite, they were not identical. Acicular ferrite with discrete cementite was identified (red arrows in Figure 3). The most significant feature of the acicular ferrite was the presence of aligned, elongated, and parallel islands of constituent martensite/austenite (M/A) or retained austenite within the prior austenite grains [19]. The yellow arrows indicate quasi-polygonal or massive ferrite phase with irregular boundaries and a few M/A constituents or retained austenite. The decomposition by tempering of the M/A constituent or the retained austenite resulted in the formation of cementite. Regarding RS (Figure 3a), besides acicular ferrite and quasi-polygonal ferrite, the analysis of other regions revealed that the austenite grain boundary structure was conserved, and in which is the substructure of fine and parallel lath ferrite (blue arrows). The lath ferrite showed different orientation depending on the austenite grain. The discrete cementite nucleated at the interface of each lath ferrite. Meanwhile, to show the microstructure, grain boundaries and the cementite distribution of RS and AS, the SEM images of RS and AS were taken, as shown in Figure 4.

**Figure 5:**
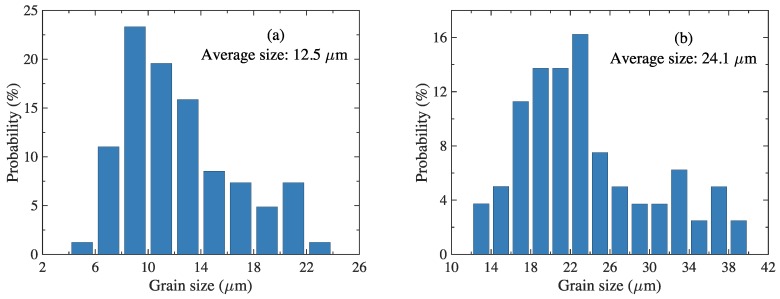
Grain size distribution of: (**a**) RS and (**b**) AS.

The statistical values of the grain sizes are shown in Figure 5. The average grain size of RS was ca. half that of AS (12.5 and 24.1 μm, respectively). Thus, the area of grain boundaries of RS was ca. 8 times larger than that of AS in a three-dimensional crystal structure.

### 3.2. Hydrogen Electrochemical Permeation Tests

**Figure 6:**
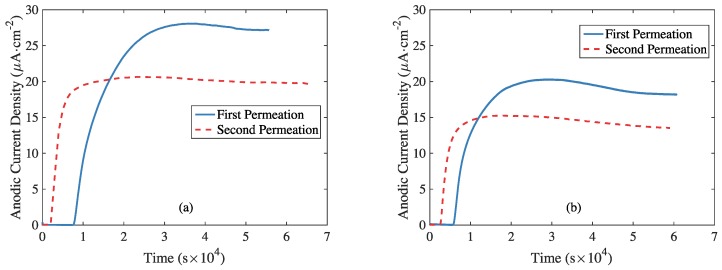
Permeation transients (first and second run) of: (**a**) RS and (**b**) AS.

Figure 6 shows the hydrogen permeation current density curves of both RS and AS. The current density increased with time after a breakthrough time before reaching a steady state value. Compared with the first permeation process, the second process showed lower breakthrough time and steady state current density values. $J_{\infty }$, $D_{eff}$, $D_{app}$, and $C_{app}$ were calculated with Equations (1)–(4) and the corresponding values are listed in Table 2.

## 4. Discussion

### 4.1. Macroscopic Characterization of Hydrogen Trap

According to Doyle´s theory [18], nickel grain boundaries are short circuit diffusion paths, resulting in apparent hydrogen diffusivities being higher than the effective hydrogen diffusivity. However, the results found herein showed the opposite trend, as shown in Table 2. Thus, the apparent hydrogen diffusivity was generally lower than the effective hydrogen diffusivity, indicating that the grain boundaries of 2.25Cr-1Mo-0.25V steel are not short circuit paths. On the contrary, the grain boundaries of 2.25Cr-1Mo-0.25V steel can be regarded as a kind of lattice defect, and it may hinder hydrogen diffusion. The grain boundaries are often denoted as hydrogen traps. The hydrogen trap density $N_{T}$ (m^−3^) can be estimated as follows [20,21,22]:

(5) $$N_{T}=\frac{C_{0}}{3}\left(\frac{D_{l}}{D_{eff}}-1\right)\times N_{A}$$

where $D_{l}$ is the lattice diffusion coefficient of hydrogen (m^2^·s^−1^, $1.28\times 10^{-4}$ m^2^·s^−1^ in ferrite [22,23] and $N_{A}$ is Avogadro’s number ($6.022\times 10^{23}$ mol^−1^). The hydrogen trap density values calculated with this expression are listed in Table 3. The values for the first permeation ($N_{T,1}$) were higher than those of the second permeation ($N_{T,2}$). This difference implies that the hydrogen traps were not completely uniform. According to previous studies [24,25], hydrogen traps can be divided into reversible and irreversible $(N_{T,re}$ and $N_{T,ir}$, respectively). Both the reversible and the irreversible hydrogen traps were empty before the first permeation. During the permeation process, hydrogen atoms occupy all types of hydrogen traps. After the permeation and once the discharge process has started, hydrogen atoms can be easily released from the reversible traps. Conversely, the irreversible traps favored hydrogen to remain adsorbed. Thus, the hydrogen trap values for the first permeation involved both reversible and irreversible traps, while the second permeation values only involved reversible hydrogen traps, as follows:

(6) $$N_{T,re}=N_{T,2}$$

(7) $$N_{T,ir}=N_{T,1}-N_{T,2}$$

**Figure 7:**
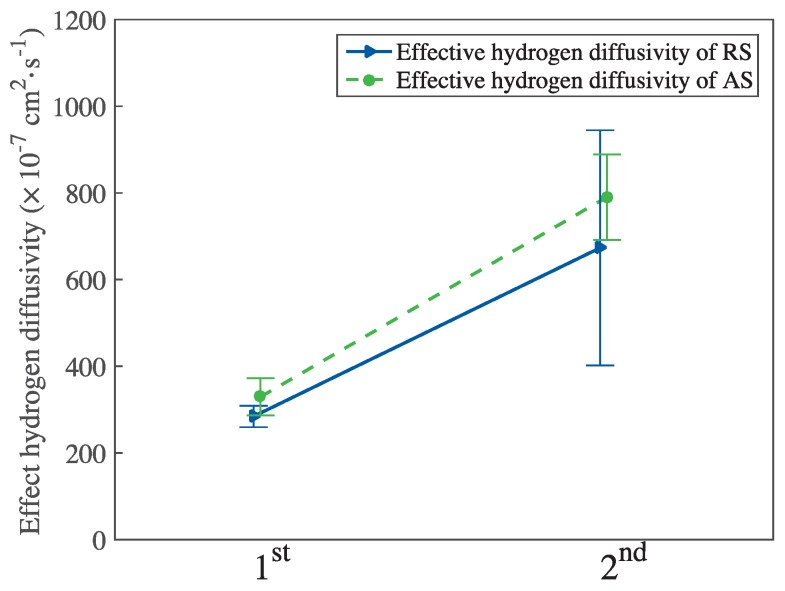
Effect of the hydrogen diffusivity of RS and AS for the first and the second permeation processes.

Table 3 shows the reversible and irreversible hydrogen trap densities for RS and AS. As shown in Table 3, more than 90% of the hydrogen was irreversibly trapped (RS 95.3%, higher than that of AS). The reversible hydrogen trap density of RS was ca. one third of that of AS. Nevertheless, the irreversible hydrogen trap density of RS was ca. 1.42 times higher than that of AS. Figure 7 summarizes the effective hydrogen diffusivity of RS and AS for the first and second permeation tests.

The effective hydrogen diffusivities of RS and AS increased by 237 and 240%, respectively, after the second permeation process. In addition, the hydrogen subsurface concentration of RS and AS decreased by 84% and 63%, respectively, after the second permeation process. These results were consistent with the hydrogen trap theory. During the first permeation process, the diffusion process was hindered because of the resistance of the irreversible hydrogen traps, resulting in lower diffusivities. Thus, a considerable amount of hydrogen atoms remained at the subsurface of the specimens in the charging cell, resulting in higher hydrogen subsurface concentrations. Conversely, during the second permeation process, higher diffusivity values and lower hydrogen subsurface concentrations were found.

The large differences between RS and AS can be explained based on the metallography results. The chemical composition and microstructure of RS were similar to those of AS. Nevertheless, both samples differed in their grain size and their ferrite microstructure. The average grain size of RS was ca. half that of RS (12.5 and 24.1 μm, respectively), which resulted in grain boundaries ca. 8 times larger than that of AS in a three-dimensional crystal structure. As for the microstructure of ferrite, RS contained larger amounts of lath ferrite than AS. Each path of lath ferrite was formed by independent nucleation via austenite transformation. The joining of two or three units of lath ferrite can be defined as a sub-boundary. Similar to the grain boundaries, massive carbon atoms can remain in the sub-boundaries region (i.e., carbon segregation). During cooling, cementite precipitates from the supersaturated ferrite, which is close to the grain boundaries and sub-boundaries regions. Thus, the different hydrogen diffusion values of RS and AS can be essentially explained in terms of their cementite content. Cementite acts as a type of hydrogen trap, hindering its diffusion. RS showed higher cementite contents, and this resulted in higher hydrogen trap density (especially the irreversible hydrogen trap density) and lower hydrogen diffusivity. Conversely, AS had a lower cementite content, resulting in lower hydrogen trap density (especially the irreversible hydrogen trap density) and higher hydrogen diffusivity.

### 4.2. Microscopic Mechanism for Hydrogen Trap

Cementite is distributed at the grain boundaries and at the interfaces of the lath ferrite, acting as important hydrogen traps. Thus, the hydrogen trap mechanism of cementite is relevant for this work. This mechanism can be explained by the following two steps. First, the diffusion thermodynamics theory was used to explain the two kinds of diffusion, namely, down-hill diffusion and up-hill diffusion (i.e., along and against the concentration gradient, respectively). Second, the Lennard–Jones potential theory was used to explain the up-hill diffusion of hydrogen from ferrite to cementite, which resulted in cementite trapping hydrogen atoms.

According to Fick’s first law applied to hydrogen diffusion:

(8) $$J_{H}=-D_{H}\frac{\partial C_{H}}{\partial x}$$

the diffusion is carried out along the hydrogen concentration gradient. When the hydrogen concentration gradient tends to zero, the flux tends to zero, and the diffusion stops. However, according to the thermodynamics, hydrogen diffusion is driven by a chemical potential gradient along a direction from regions of high chemical potential to regions of low chemical potential. In most cases, the concentration gradient follows the same trend as the chemical potential gradient, although this is not an absolute rule. In contrast, the diffusion process, described by thermodynamics, is more universal. At constant temperature and pressure, the essential of a diffusion process is that the change of the Gibbs free energy in a solid solution is less than zero, ΔG<0. Thus, in the hydrogen diffusion process, the driving force for the diffusion of hydrogen atoms $F_{H}$ along the x-direction can be determined by:

(9) $$F_{H}=-\frac{\partial \mu_{H}}{\partial x}$$

where $\mu_{H}$ is the chemical potential of hydrogen. The diffusion rate $v_{H}$ is proportional to $F_{H}$:

(10) $$v_{H}=B_{H}F_{H}$$

where the proportional coefficient $B_{H}$ is defined as the mobility, which is related to the moving resistance. Thus, the hydrogen flux $J_{H}$ is:

(11) $$J_{H}=C_{H}v_{H}$$

where $C_{H}$ is the hydrogen concentration. According to Equations (8)–(11):

(12) $$J_{H}=-C_{H}B_{H}\frac{\partial \mu_{H}}{\partial x}$$

In a non-ideal solid solution, the chemical potential of hydrogen can be determined by:

(13) $$\mu_{H}=\mu_{H}^{0}+RT\cdot ln(\gamma_{H}x_{H})$$

where $\mu_{H}^{0}$ is defined as the chemical potential of pure hydrogen, R is the gas constant, and $\gamma_{H}$ is the activity coefficient, which is used to correct the deviation from ideality, as predicted by the Raoult’s law. If the activity coefficient is less than 1, deviation from the Raoult’s law is negative, which implies that the components present attractive forces. On the contrary, if the activity coefficient is higher than 1, deviation from the Raoult’s law is positive, and the components present repellent forces [26]. $x_{H}$ is the mole fraction of hydrogen:

(14) $$x_{H}=\frac{C_{H}}{\sum C_{i}}$$

where $\sum C_{i}$ is a constant. Then, the flux can be simply calculated as:

(15) $$J_{H}=-B_{H}RT\left(1+\frac{\partial ln\gamma_{H}}{\partial lnx_{H}}\right)\frac{\partial C_{H}}{\partial x}$$

As shown in Equation (8), compared with Fick’s first law, the diffusivity of hydrogen can be calculated as:

(16) $$D_{H}=B_{H}RT\left(1+\frac{\partial ln\gamma_{H}}{\partial lnx_{H}}\right)$$

Therefore, the sign of the diffusivity depends on the factor $\left(1+\frac{\partial ln\gamma_{H}}{\partial lnx_{H}}\right)$. If $\left(1+\frac{\partial ln\gamma_{H}}{\partial lnx_{H}}\right)>0$, diffusion takes place along the concentration gradient, from a region of high concentration to a region of low concentration. This is called down-hill diffusion. If $\left(1+\frac{\partial ln\gamma_{H}}{\partial lnx_{H}}\right)<0$, diffusion takes place along the fixed direction, regardless the concentration gradient. Thus, the concentration of the region with low chemical potential increases at the expense of those of other regions. This is called up-hill diffusion.

**Figure 8:**
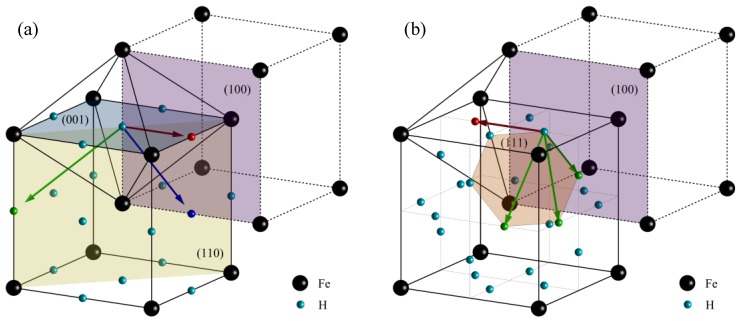
Schematic of the solute interstitial hydrogen atoms in the ferrite crystal cells in: (**a**) octahedral sites and (**b**) tetrahedral sites.

Since hydrogen is significantly smaller than iron, solute hydrogen atoms are located at the interstitial sites of the ferrite crystal. As shown in Figure 8, there are two types of interstitial sites, namely, octahedral sites and tetrahedral sites. Hydrogen atoms placed at the same type of interstitial sites are equal. The arrows show hydrogen diffusion direction through the (001), (100), and (110) crystallographic planes in an octahedral sites solid solution and through the (111) and (100) crystallographic planes in a tetrahedral sites solid solution.

**Figure 9:**
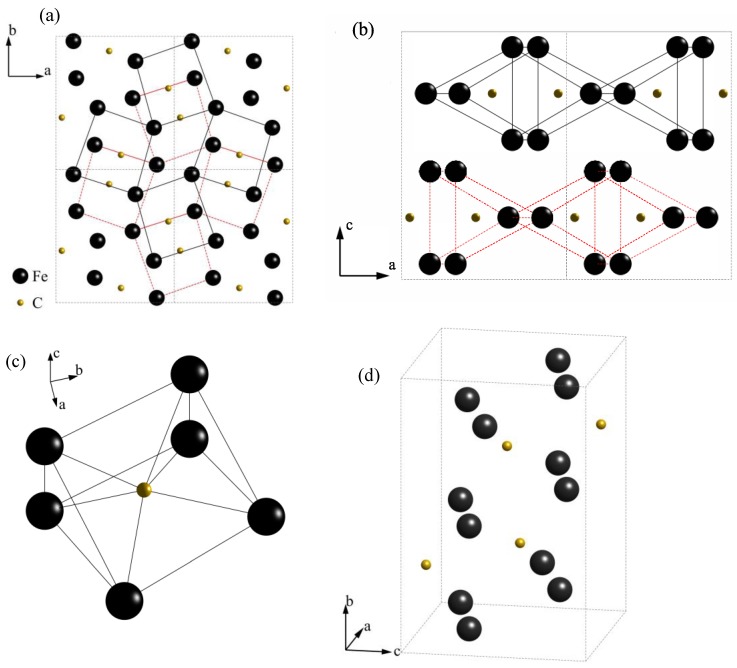
Schematic of the crystal structure of cementite: the structure of the supercell from the projection of: (**a**) (001) plane and (**b**) (010) plane; (**c**) structure of the trigonal prism and (**d**) structure of a cementite crystal cell.

Figure 9a,b show the projections of a 2×2 supercell of cementite in the (001) and (010) crystallographic planes. As shown in Figure 9b, cementite has a layered structure, with each layer consisting of identical and continuous trigonal prisms (highlighted with a black solid line for the first layer and a red dashed line for the second layer). Each trigonal prism consists of 6 Fe atoms and 1 C atom at the corners and center, respectively, as shown in Figure 9c. Figure 9d shows an orthorhombic (space group Pnma) crystal cell of cementite. However, the position of hydrogen in cementite remains unclear. Thus, it can be assumed that hydrogen atoms are located at the regions having the largest interstices.

Since the iron crystal remains unchanged upon diffusion of hydrogen atoms, the multi-compound system formed by hydrogen and steel can be considered a solid solution. Thus, it can be assumed that there are no chemical bonds between hydrogen and other atoms in steel, such that they exist independently. The hydrogen atoms and iron atoms are nonpolar such that the Lennard–Jones potential [14] can be used to determine the interatomic forces. The Lennard–Jones potential theory explains the interaction between a pair of neutral atoms or molecules. In the crystal, which is formed by inert gases atoms, the interatomic van der Waals force is successfully described by the Lennard–Jones potential theory, and the calculated values of lattice constant extremely approximate to the experimental values. The most common expression of the Lennard–Jones potential is:

(17) $$u\left(r\right)=4\varepsilon \left[{\left(\frac{\sigma }{r}\right)}^{12}-{\left(\frac{\sigma }{r}\right)}^{6}\right]$$

where ε is the depth of the potential well, σ is the finite distance at which the interatomic potential is zero, and r is the distance between the atoms. The force between two atoms is:

(18) $$F\left(r\right)=\frac{du}{dr}=24\varepsilon \left(-2\sigma^{12}r^{-13}+\sigma^{6}r^{-7}\right)$$

Regarding a crystal with N atoms, the total potential can be expressed as:

(19) $$U=\frac{N}{2}\overset{N}{\underset{j}{\sum }}u\left(r_{ij}\right)$$

Considering R as the distance between the two closest atoms, then $r_{ij}=a_{j}R$. Equation (19) can be simplified to:

(20) $$U\left(R\right)=2N\varepsilon \left[A_{12}{\left(\frac{\sigma }{R}\right)}^{12}-A_{6}{\left(\frac{\sigma }{R}\right)}^{6}\right]$$

where,

(21) $$A_{12}=\overset{N}{\underset{j}{\sum }}a_{j}^{-12},\;A_{6}=\overset{N}{\underset{j}{\sum }}a_{j}^{-6}$$

The balanced interatomic distance $R_{0}$, which is also the lattice constant, can be determined as follows:

(22) $${\left(\frac{\partial U}{\partial R}\right)}_{R_{0}}={\left\{\frac{2N\varepsilon }{R}\left[-12A_{12}{\left(\frac{\sigma }{R}\right)}^{12}+6A_{6}{\left(\frac{\sigma }{R}\right)}^{6}\right]\right\}}_{R_{0}}=0$$

then σ can be calculated as:

(23) $$\sigma =R_{0}{\left(\frac{2A_{12}}{A_{6}}\right)}^{-\frac{1}{6}}$$

and ε can be determined with the bulk modulus K:

(24) $$K={\left(\frac{\partial^{2}U}{\partial V^{2}}\right)}_{V_{0}}V_{0}=\frac{R_{0}^{2}}{9V_{0}}{\left(\frac{\partial^{2}U}{\partial R^{2}}\right)}_{R_{0}}=\frac{4\varepsilon }{\sigma^{3}}A_{12}{\left(\frac{A_{6}}{A_{12}}\right)}^{\frac{5}{2}}$$

which can be simplified to:

(25) $$\varepsilon =\frac{1}{4}K\sigma^{3}A_{6}^{-\frac{5}{2}}A_{12}^{\frac{3}{2}}$$

The bulk modulus in Equation (25) can be determined experimentally or by theoretical calculations. The bulk modulus of ferrite uses the experimental results of J.A. Rayne [27], and is determined as:

(26) $$K=\frac{E}{3\left(1-2\mu \right)}$$

where E is the Young’s modulus and μ is the Poisson ratio. The bulk modulus of cementite has not been determined experimentally yet. Table 4 shows the elastic moduli calculated from first principle calculations by different researchers [28,29,30,31].

The bulk modulus can be determined by the Voigt–Reuss–Hill method [32]:

(27) $$K=\frac{1}{3}\left(C_{11}+2C_{12}\right)$$

The *C*_11_ and *C*_12_ values calculated by different researchers were similar (Table 4). Thus, it is reasonable to select arbitrary theoretically calculated values as true elastic moduli values of cementite. In this study, we selected the values from Jiang [28] to calculate the Lennard–Jones potential. The lattice constant and bulk modulus of ferrite and cementite are summarized in Table 5.

**Figure 10:**
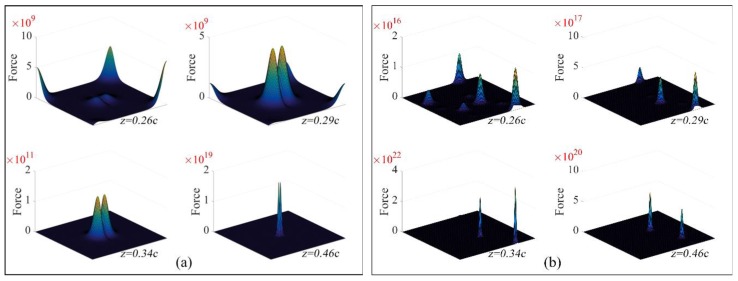
Force of a hydrogen atom at different positions in: (**a**) a ferrite crystal cell and (**b**) a cementite crystal cell.

A 3×3×3 supercell was used as a model for calculating the Lennard–Jones potential and force. The hydrogen was located in the center cell. The force between one hydrogen atom and another atom can be calculated by Equation (18). The vector sum of the force can be qualitatively considered the force of the hydrogen atom in ferrite or cementite. Table 6 shows the results of these calculations, and Figure 10 shows the force of a hydrogen atom on random layers of the ferrite and cementite crystal cells. It is obvious that the force of a hydrogen atom is significantly larger in cementite than that in ferrite. Thus, cementite has a stronger attractive force towards hydrogen atoms and therefore the activity coefficient $\gamma_{H}$ of cementite is lower than that of ferrite. We considered that the direction of hydrogen diffusion from ferrite to cementite is positive. According to Equation (16), if the concentration of hydrogen in cementite is lower, the diffusivity is greater than zero, $D_{H}>0$. Also, hydrogen atoms diffuse along the concentration gradient. If the concentration of hydrogen in cementite is higher, the diffusivity is less than zero, $D_{H}<0$. The direction of diffusion is opposite to the hydrogen concentration gradient (i.e., up-hill diffusion). Then, the concentration of hydrogen in cementite increased continuously. Furthermore, hydrogen atoms trapped by cementite are hardly released (i.e., a high activation energy must be overcome). This is the hydrogen trap mechanism of cementite.

## 5. Conclusions

In this work, the hydrogen diffusivity and trap characteristics of heat-treated (annealed) and untreated 2.25Cr-1Mo-0.25V steels were studied using an electrochemical permeation method. The mechanism of cementite on the hydrogen diffusion and trap was studied using thermodynamics-based and Lennard–Jones potential theories. The main findings of the present study were:

1. According to the electrochemical permeation test results, cementite located at the grain boundaries and at the interfaces of lath ferrite acted as a kind of hydrogen trap, mostly as an irreversible hydrogen trap.
2. According to the diffusion thermodynamics and the Lennard–Jones potential theories, the interaction between hydrogen atoms and other atoms in cementite is larger than that in ferrite, thus, hydrogen diffusion from ferrite to cementite is up-hill diffusion and hydrogen atoms will be collected around the cementite, making the cementite a hydrogen trap.

## Figures and Tables

**Table 1:** Chemical composition of 2.25Cr-1Mo-0.25V steel (wt %) [2].

Element	C	Si	Mn	P	S	Cr	Mo	V	Al
Percentage	0.15	0.1	0.54	0.009	0.01	2.3	0.98	0.3	0.05

**Table 2:** Results of the hydrogen permeation experiments.

Material	Time of Experiment	Hydrogen Flux $J_{\infty }$ (10^−11^ mol·m^−2^·s^−1^)	Effective Hydrogen Diffusivity $D_{eff}$ (10^−7^ cm^2^·s^−1^)	Apparent Hydrogen Diffusivity $D_{app}$ (10^−7^ cm^2^·s^−1^)	Subsurface Hydrogen Concentration $C_{0}$ (10^−5^ mol·cm^−3^)
RS	1st	30.84	284.36	201.37	1.63
	2nd	13.27	673.39	578.03	0.27
AS	1st	28.34	329.38	255.18	1.36
	2nd	25.48	790.27	660.94	0.51

**Table 3:** Hydrogen trap density for RS and AS (10^18^ m^−3^).

Material	Hydrogen Trap Density for 1st Permeation $N_{T,1}$	Hydrogen Trap Density for 2nd Permeation $N_{T,2}$	Reversible Hydrogen Trap Density $N_{T,re}$	Irreversible Hydrogen Trap Density $N_{T,ir}$
RS	11.62	0.55	0.55	11.07
AS	8.58	0.77	0.77	7.81

**Table 4:** Elastic moduli of cementite calculated from first principle calculations by different researchers.

Research Scholars	Method	*C*_11_	*C*_22_	*C*_33_	*C*_12_	*C*_23_	*C*_13_	*C*_44_	*C*_55_	*C*_66_	*E*	*B*
Jiang et al. [28]	Relaxed, energy-strain	388	345	322	156	162	164	15	134	134	224	249
	Relaxed, stress-strain	395	347	325	158	163	169	18	134	135	227	252
Nikolussi et al. [29]	Relaxed, stress-strain	385	341	316	157	167	162	13	131	131	224	243
Lv et al. [30]	Relaxed, stress-strain	393	340	319	144	149	141	-60	145	118	213	218
Henriksson et al. [31]	Unrelaxed, energy-strain	394	412	360	157	166	146	83	133	136	234	301

**Table 5:** Lattice constant and bulk modulus of ferrite and cementite.

Structure	Cell Geometries	Lattice Constant (nm) [33]			Bulk Modulus (GPa)
		a	b	c	
Ferrite	BCC	0.2863	0.2863	0.2863	173.4 [27]
Cementite	Orthorhombic	0.5038	0.6727	0.4484	231.1 [28]

**Table 6:** Results of the Lennard–Jones force calculation for ferrite and cementite.

Microstructure	Octahedral Sites	Tetrahedral Sites	Random Position		
			Maximum	Minimum	Average
Ferrite	$2.1970\times 10^{5}$	$4.0481\times 10^{4}$	$2.2136\times 10^{43}$	6.3449	$1.8049\times 10^{37}$
Cementite	-	-	$1.4975\times 10^{47}$	$8.1101\times 10^{6}$	$2.9719\times 10^{40}$
